# Viral expression directs the fate of B cells in BLV-infected sheep

**DOI:** 10.1186/1742-4690-8-S1-A3

**Published:** 2011-06-06

**Authors:** Arnaud Florins, Amel-Baya Bouzar, Alix Debrogniez, Carole François, Michal Reichert, Luc Willems

**Affiliations:** 1 Cellular and molecular biology, Gembloux Agro-Bio Tech, University of Liège, Gembloux, Belgium; 2 National Veterinary Research Institute, Pulawy, Poland; 3 Interdisciplinary Cluster for Applied Genoproteomics, University of Liège, Liège, Belgium

## 

There is a long lasting debate about the latency of human T-lymphotropic virus type 1 (HTLV-1) and bovine leukemia virus (BLV). Evidence indicates that these viruses are transcriptionally silent and replicate through mitotic division of infected cells (clonal expansion). However, this model is inconsistent with the permanent and vigorous stimulation of the host immune response directed against these viruses.

To address this apparent paradox, we studied the fate of cells in which viral expression was transiently induced. Using a dual fluorochrome labeling approach, we show that virus-positive and negative cell populations have different kinetics in BLV-infected sheep. Furthermore, cyclosporine treatment completely abrogates the difference in kinetics, consistent with a role of the immune response in controlling virus expressing cells.

